# Age-related effects of comprehensive in-patient physiotherapy after lung cancer surgery across age groups: an ambispective observational cohort study in middle-aged and older patients

**DOI:** 10.1186/s12890-026-04592-6

**Published:** 2026-08-13

**Authors:** Ayse Sena Manzak Dursun, Betul Cinar, Fuat Gökdemir, Omer Soysal, Alis Kostanoğlu

**Affiliations:** 1 Department of Physiotherapy and Rehabilitation, Faculty of Health Sciences, Bezmialem Vakif University, İstanbul, 34050 Turkey; 2 Department of Thoracic Surgery, Medical Faculty, Bezmialem Vakif University, İstanbul, Türkiye

**Keywords:** Lung cancer surgery, Pulmonary rehabilitation, Physiotherapy, Length of stay, Inflammatory biomarkers, Aging

## Abstract

**Background:**

Postoperative recovery after lung cancer surgery may be influenced by age, comorbidity burden, and perioperative rehabilitation strategies. However, evidence regarding the age-related effects of structured early inpatient physiotherapy programs remains limited. This study aimed to evaluate the age-related effects of a comprehensive in patient physiotherapy (CIPT) program on hospital length of stay (LoS), clinical parameters, symptom-related outcomes, and inflammatory biomarkers in patients undergoing lung cancer surgery.

**Methods:**

This ambispective observational cohort study included patients who underwent lung cancer surgery and received a structured CIPT program during the postoperative inpatient period as part of standard postoperative care. Participants were categorized into middle-aged and older groups. Outcomes included hospital LoS, clinical parameters (blood pressure, resting heart rate, oxygen saturation, and respiratory rate), symptom-related outcomes (dyspnea and fatigue with Modified Borg Scale, and incisional pain with Visual analog scale), and inflammatory biomarkers (C-reactive protein (CRP), neutrophil-to-lymphocyte ratio (NLR), and albumin). Pre- and post-program changes within groups and differences between age groups were analyzed.

**Results:**

Median LoS was 7 [5–10] days in the middle-aged group and 7 [6–10] days in the older group, with no significant difference between groups. Significant improvements in resting heart rate, oxygen saturation, and dyspnea were observed in the older group, whereas dyspnea, fatigue, and incisional pain improved significantly in the middle-aged group. No significant differences were detected between age groups in clinical parameters or symptom-related outcomes. CRP and NLR levels decreased significantly in both groups, while albumin showed a non-significant upward trend over time. Despite a higher comorbidity burden in the older group, no adverse events or clinical instability were observed during the rehabilitation program.

**Conclusion:**

The CIPT program appears to be safe and feasible for both middle-aged and older patients following lung cancer surgery. Similar outcomes between age groups suggest that age alone may not represent a barrier to participation in structured early inpatient rehabilitation programs. Early physiotherapy integrated into postoperative care pathways may facilitate recovery without increasing hospital length of stay across age groups, which may also help reduce the potential economic burden associated with prolonged hospitalization.

**Trial registration:**

ClinicalTrials.gov, NCT07441863. First submitted on 17 February 2026 and first posted on 02 March 2026 retrospectively registered.

## Introduction

Lung cancer remains a major public health problem at the center of the global cancer burden. According to GLOBOCAN 2022 estimates, lung cancer is the most frequently diagnosed cancer worldwide and the leading cause of cancer-related mortality [1]. Although lung cancer continues to have a relatively poor prognosis in terms of survival, European cancer registry data indicate a modest improvement in lung cancer survival over time [2]. Expanded screening programs, advances in diagnostic techniques, and the development of targeted therapeutic strategies have contributed to this increase in survival rates [3]. In addition to improvements in survival, the global aging of the population has shifted the epidemiological profile of lung cancer toward an increasingly older patient population [4].

Surgical resection remains the gold standard treatment with the highest potential for cure in lung cancer [5]. However, lung resection is associated with a considerable risk of postoperative complications, particularly pulmonary complications in the early postoperative period [6]. Older individuals are at greater risk for postoperative pulmonary complications (PPC), independent of the additional contribution of comorbidities commonly present in this population. PPC are strongly associated with increased healthcare costs, prolonged hospital stays, and higher morbidity and mortality rates [7–9]. Therefore, the implementation of physiotherapy programs plays a crucial role in improving clinical outcomes and preventing postoperative complications in this population [10].

Postoperative physiotherapy programs (PPP) are recommended by the European Society of Thoracic Surgeons, the European Respiratory Society, and the American College of Chest Physicians. These programs are considered an essential component of enhanced recovery protocols aimed at reducing postoperative symptom burden, accelerating functional recovery, and minimizing the hospital length of stay (LoS) [10–12]. Such programs may include one or more interventions such as pain management strategies, positioning, airway clearance techniques, breathing exercises, early mobilization, postural exercises, and exercises to maintain normal joint range of motion [10, 13]. Nevertheless, evidence suggests that structured, multi-component rehabilitation programs provide more consistent and substantial benefits than single-component interventions [12].

Despite the well-established benefits of PPP following lung cancer surgery, the effectiveness of these programs across different age groups remains unclear in the current literature. Therefore, the aim of the present study was to compare the LoS, clinical parameters, symptom-related outcomes, and inflammatory biomarkers between middle-aged and older patients who received a comprehensive in-patient physiotherapy (CIPT) program after lung cancer surgery.

## Methods

### Study design and participants

This study was designed as an ambispective observational cohort study and conducted at Bezmialem Vakif University Hospital. The study included patients receiving routine comprehensive in-patient physiotherapy following lung cancer surgery as part of standard postoperative care. Ethical approval was obtained from the Bezmialem Vakif University Scientific Research Ethics Committee (No: 2026/72) in accordance with the principles of the Declaration of Helsinki. The study was retrospectively registered at ClinicalTrials.gov (NCT07441863; first submitted on 17 February 2026 and first posted on 02 March 2026). All evaluated outcomes represented routinely documented postoperative clinical variables, and written informed consent for the anonymous use of clinical data was routinely obtained from all patients in accordance with institutional procedures.

Participants were recruited according to the following inclusion criteria: age between 45 and 90 years; having undergone open thoracotomy (lobectomy, or pneumonectomy) and video-assisted thoracoscopic surgery (VATS; wedge resection) for lung cancer; being hemodynamically stable during the early postoperative period; and providing written informed consent to participate in a structured, comprehensive in-patient physiotherapy program. Patients were excluded if they had another active malignancy, hemodynamic instability restricting mobilization, severe cognitive impairment limiting cooperation, neurological or orthopedic conditions affecting mobility, long-term mechanical ventilation requirements, physician-determined contraindications for physiotherapy, prolonged intensive care unit stay, postoperative pulmonary complications, or hospitalization exceeding 30 days.

**Fig. 1:**
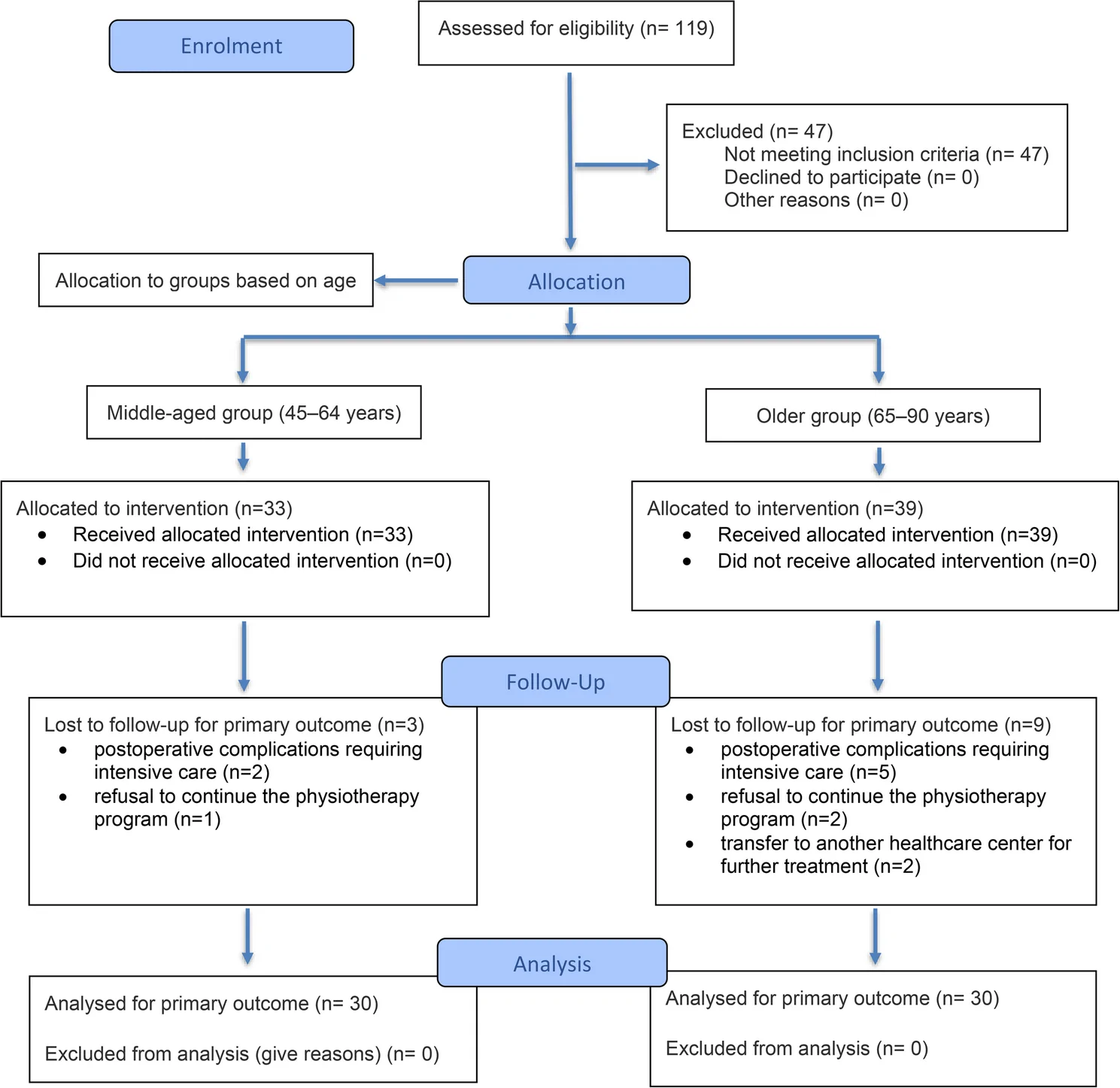
Participant Flow Diagram

All participants received detailed information regarding the study procedures, and written informed consent was obtained prior to enrollment. Patients included in the study were stratified into two age groups for comparative analyses: middle-aged (45–64 years) and older (65–90 years) (Fig. 1).

Patients who underwent lung resection surgery received the CIPT program starting from the first day of their transfer to the thoracic surgery ward and continuing until hospital discharge. Baseline assessments were performed prior to the initiation of the CIPT program. Final evaluations were conducted on the day of discharge before the CIPT session to avoid the potential acute effects of the intervention on the measurements.

### Outcome measures

The primary outcome measure of the study was the LoS, as hospital discharge is primarily determined by patients’ overall clinical stability [14]. Secondary outcomes included clinical parameters, symptom-related outcomes, and inflammatory biomarkers.

LoS was calculated as the time interval from admission to the thoracic surgery ward to the day of hospital discharge.

Clinical parameters such as peripheral oxygen saturation (SpO₂, %), systolic and diastolic blood pressure (SBP and DBP, mmHg), resting heart rate (HR_rest_, bpm), and respiratory rate (RR, breaths/min) were measured. Symptom-related outcomes such as dyspnea and fatigue were assessed using the Modified Borg Scale, and pain intensity was evaluated using the Visual Analog Scale (VAS), both of which are previously published and widely validated instruments [15, 16].

Inflammatory biomarkers were obtained from blood samples and included C-reactive protein (CRP), neutrophil-to-lymphocyte ratio (NLR), and albumin levels.

## Intervention

Patients received standard postoperative care in the intensive care unit (ICU). Following transfer from the ICU to the thoracic surgery ward, they participated in the CIPT program, which was initiated on the first day of ward admission and continued until hospital discharge.

The CIPT program consisted of patient education, chest physiotherapy, postural exercises, normal range-of-motion (ROM) exercises for the upper and lower extremities, and early mobilization. The detailed structure and components of the CIPT program are presented in Table 1.

**Table 1:** Structure of the CIPT program

Component	Intervention and Dosage / Progression
Patient Education	Incision support during coughing using hands or pillow; cardiac positioning
Breathing Exercises	Low-frequency breathing, deep diaphragmatic breathing, segmental breathing, sustained maximal inspiration (2 sets × 5 repetitions)
Incentive Spirometry	Incentive spirometer training (2 sets × 5 repetitions)
Airway Clearance	Forced expiration technique (2–3 times at the end of the chest physiotherapy)
Early Mobilization	Progressive mobilization from bedside and in-room walking to corridor ambulation up to 100 m
Postural Exercises	Scapulohumeral mobilization, pectoral stretching, chin-tuck (2 sets × 5 repetitions)
Lower Extremity ROM	Ankle pumping, knee flexion–extension, hip flexion–extension, hip abduction–adduction (2 sets × 5 repetitions)
Upper Extremity ROM	Hand pumping, elbow flexion–extension, shoulder flexion up to 80° (2 sets × 5 repetitions)

Patient education focused on incision support during coughing using the hands or a pillow to reduce incisional pain and appropriate therapeutic positioning, including the cardiac position, to facilitate breathing.

Chest physiotherapy, postural exercises, and ROM exercises were performed in two sets of five repetitions. Early mobilization progressively advanced from in-room mobilization to corridor ambulation up to 300 m depending on patient tolerance. The program was conducted daily during hospitalization, including one supervised session with a physiotherapist and two additional sessions performed independently by the patient or with the assistance of a caregiver.

All exercises were performed under continuous pulse oximetry monitoring, and standard safe exercise principles were followed throughout the intervention.

### Statistical analysis

The statistical analysis of all data was conducted using IBM SPSS Statistics software (SPSS Inc., USA). The normality of the data distribution was assessed using the Shapiro–Wilk test. Categorical variables were compared between groups using the χ² test. Independent samples t-test or Mann–Whitney U test was used for between-group comparisons depending on data distribution.

To evaluate the effects of time and group on the outcome variables, a mixed-design repeated measures ANOVA was performed, with time as the within-subject factor and group as the between-subject factor. Since the within-subject factor (time) consisted of only two levels (baseline and post-training), the assumption of sphericity was inherently satisfied; therefore, Mauchly’s test of sphericity and related corrections (Greenhouse–Geisser and Huynh–Feldt) were not required. Planned pairwise comparisons were performed for all variables to identify significant intragroup changes from baseline to post-training within each group separately. For these comparisons, Bonferroni correction was applied to account for multiple testing.

A significance level of *p* < 0.05 was considered statistically significant for all analyses. Complementarily, the effect size was calculated using partial eta-squared (ηp²), with small, medium, and large effect sizes classified as 0.01, 0.06, and 0.14, respectively [17].

The sample size was calculated based on the primary outcome, hospital length of stay. Based on a recent study investigating the effects of early mobilization and enhanced recovery protocols after lung resection, the standard deviation (SD) of LoS was observed to be approximately 1.39 days [18]. To ensure a conservative estimate, the expected SD was set at 1.5 days. To detect a minimal clinically important difference of 1.2 days in LoS between the middle-aged and older groups, with an alpha error of 0.05 and a statistical power of 80%, a minimum of 25 patients per group was required. Accounting for an estimated 20% attrition and missing data rate, the final sample size was established at 30 patients per group, yielding a total sample of 60 patients.

## Results

A total of one hundred and nineteen patients were assessed for eligibility. Seventy-two patients who met the inclusion criteria were enrolled in the study and allocated into two groups according to age. During the study period, twelve patients dropped out for various reasons, including postoperative complications requiring intensive care (middle-aged group *n* = 2; older group *n* = 5), refusal to continue the physiotherapy program (*n* = 3), and transfer to another healthcare center for further treatment (*n* = 2).

60 patients (30 middle-aged, 30 older ) completed the study. The flow of participants throughout the study is illustrated in Fig. 1.

The comparison of the demographic and clinical characteristics of the patients between the groups is presented in Table 2. There were no significant differences between the groups in terms of demographic and clinical characteristics, except for comorbidities (*p* > 0.05). Comorbidities were more frequent in the older group and included chronic obstructive pulmonary disease, hypertension, diabetes mellitus, coronary artery disease, and chronic kidney failure.

**Table 2:** Comparison of baseline values of demographic and clinical characteristics of middle-aged and older groups

	Middle-aged Group (*n* = 30)	Older Group (*n* = 30)	Intergroup Differences (*p*)
Age (years)	53,67 ± 5,79	71,47 ± 6,04	**< 0,001**
Gender, n (%)			
Female	12 (%40)	6 (%20)	0,133
Male	18 (%60)	24 (%80)	
Body Mass Index (kg/m2)	26,96 ± 5,22	27,08 ± 5,65	0,934
Comorbidities (n)	1,40 ± 1,22	2,20 ± 1,51	**0**,**028**
Smoking History, n (%)			
Positive	14 (46,66)	17 (%56,66)	0,447
Negative	16 (%53,34)	13 (%43,34)	
Type of Surgery, n (%)			
Pneumonectomy	6 (%20)	7 (%23,3)	0,937
Lobectomy	10 (%33,3)	9 (%30)	
Wedge Resection (VATS)	14 (%46,7)	14 (46,7)	
Length of ICU Stay (day)	1,40 ± 1,22	2,20 ± 1,51	0,256

Data are presented as mean±Standard deviation or n (%)

*VATS* Video-Assisted Thoracoscopic Surgery

significance at *p *< 0,05

Baseline values of the clinical parameters, symptom-related outcomes, and inflammatory biomarkers showed no significant differences between the groups (Table 3).

**Table 3:** Comparison of the baseline values in clinical parameters, symptom-related outcomes parameters, and inflammatory biomarkers between middle-aged and older groups

	Middle-Aged Group (*n* = 30)	Older Group (*n* = 30)	Intergroup Differences *p* (%95 CI)
Clinical Parameters			
SBP (mmHg)	117,67 ± 12,507	123,40 ± 17,13	*p* = 0,145 (-2,020–13,48)
DBP (mmHg)	74,27 ± 13,27	72,20 ± 12,12	*p* = 0,531 (-8,63–4,50)
HR_rest_ (bpm)	93,53 ± 16,91	91,53 ± 16,77	*p* = 0,647 (-10,70–6,70)
SpO_2_ (%)	93,27 ± 14,02	94,30 ± 2,84	*p* = 0,695 (-4,29–6,36)
RR (breaths/min)	24,17 ± 7,20	24,23 ± 6,78	*p* = 0,971 (-3,55–3,68)
Symptom-Related Outcomes			
Dyspnea (modified Borg scale)	2,00 ± 2,44	2,53 ± 2,76	*p* = 0,432 (-0,81–1,88)
Fatigue (modified Borg scale)	3,40 ± 2,58	3,33 ± 2,60	*p* = 0,921 (-1,40–1,27)
Pain (VAS)	4,50 ± 2,89	3,60 ± 2,29	*p* = 0,188 (-2,252–0,45)
Inflammatory Biomarkers			
CRP (mg/L)	141,14 ± 86,05	145,64 ± 75,05	*p* = 0,830 (-37,22–46,23)
NLR	10,20 ± 9,96	11,75 ± 9,28	*p* = 0,536 (-3,42–6,52)
Albumin (g/dL)	6,17 ± 8,43	7,86 ± 10,75	*p* = 0,501 (-3,30–6,68)

Data are presented as mean±standard deviation or n (%)

*SBP*Systolic blood pressure, *DBP* Diastolic blood pressure, *HR*_rest_ Resting heart rate, *SpO2* Oxygen saturation, *RR* Respiratory rate, *VAS* Visual Analog Scale, *CRP* C-Reactive Protein, *NLR* Neutrophil-to-lymphocyte ratio

**Fig. 2:**
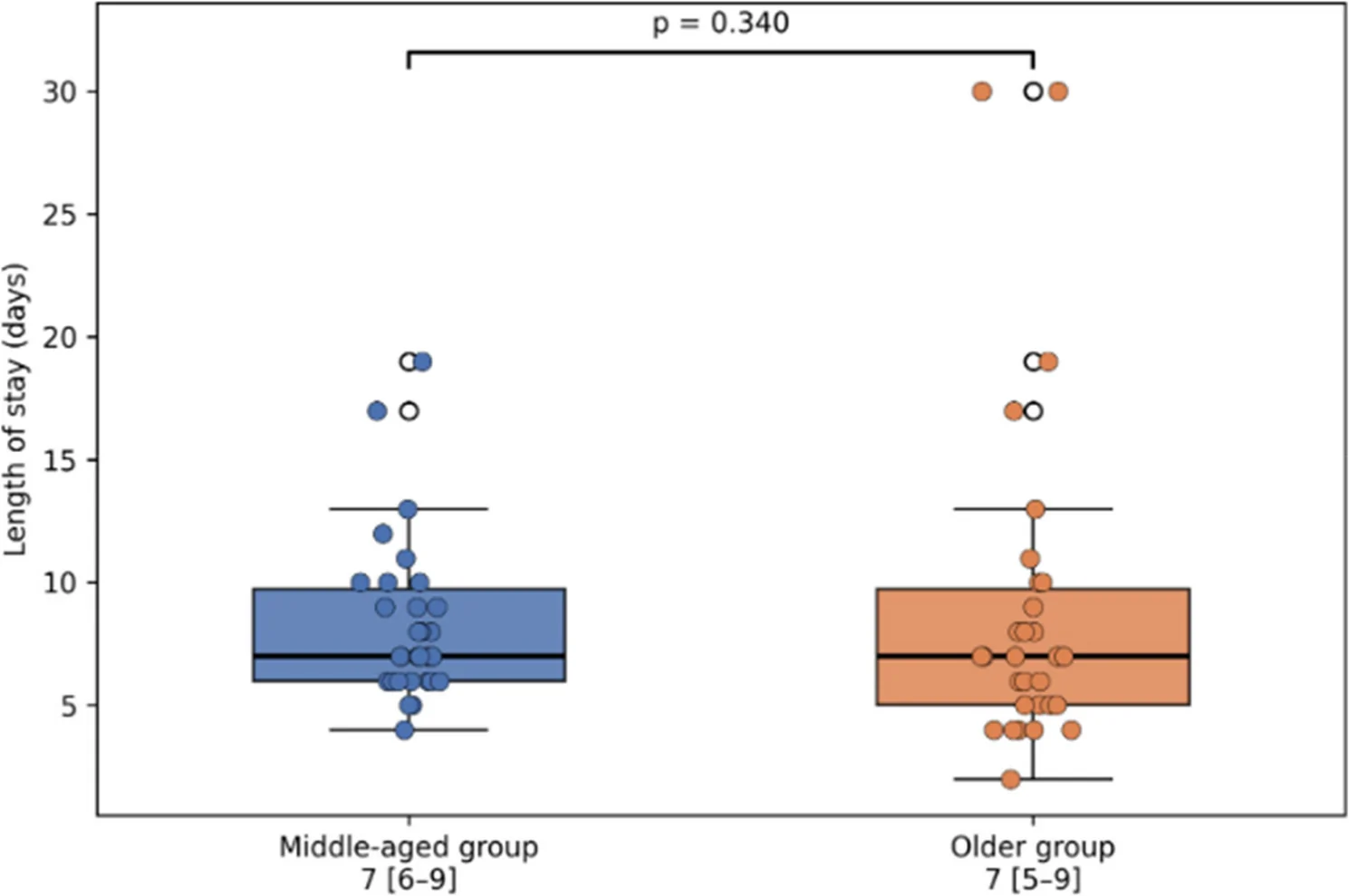
Hospital length of stay by age group

The length of hospital stay for both groups is presented in Fig. 2. There was no significant difference in LoS between the groups, with a median LoS of 7 days in both groups.

The intergroup and intragroup comparisons of clinical parameters, symptom-related outcomes, and inflammatory biomarkers before and after the CIPT program are presented in Table 4. No significant time × group interaction effect was observed for any of the parameters.

**Table 4:** Comparison of clinical parameters, symptom-related clinical parameters, and inflammatory biomarkers before and after the CIPT program between middle-aged and older groups

	Middle-aged Group (*n* = 30)			Older Group (*n* = 30)			Intergroup Differences**		
	Baseline	Post-training	Intragroup Differences*	Baseline	Post-training	Intragroup Differences*	Time	Group	Time x Group
Clinical Parameters									
SBP (mmHg)	117,67 ± 12,50	113,50 ± 11,829	*p* = 0,305	123,40 ± 17,13	120,83 ± 24,143	*p* = 0,526	*p* = 0,242 η2 = 0,024 f = 1,400	*p* = 0,058 η2 = 0,060 f = 3,727	*p* = 0,780 η2 = 0,052 f = 0,079
DBP (mmHg)	74,27 ± 13,27	73,50 ± 10,09	*p* = 0,721	72,20 ± 12,12	72,83 ± 7,95	*p* = 0,768	*p* = 0,965 η2 < 0,001 f = 0,002	*p* = 0,574 η2 = 0,005 f = 0,319	*p* = 0,645 η2 = 0,004 f = 0,215
HR_rest_ (bpm)	93,53 ± 16,91	93,23 ± 14,48	*p* = 0,916	91,53 ± 16,77	84,93 ± 15,24	*p* = 0,**023**	*p* = 0,089 η2 = 0,049 f = 2,985	*p* = 0,156 η2 = 0,034 f = 2,065	*p* = 0,120 η2 = 0,041 f = 2,488
SpO_2_ (%)	93,27 ± 14,02	94,00 ± 14,04	*p* = 0,089	94,30 ± 2,84	95,37 ± 2,39	*p* = 0,**015**	*p* = 0,**004** η2 = 0,134 f = 8,985	*p* = 0,645 η2 = 0,004 f = 0,215	*p* = 0,581 η2 = 0,005 f = 0,308
RR (breaths/min)	24,17 ± 7,20	22,80 ± 7,65	*p* = 0,274	24,23 ± 6,78	22,90 ± 6,66	*p* = 0,286	*p* = 0,129 η2 = 0,039 f = 2,376	*P* = 0,959 η2 < 0,001 f < 0,001	*p* = 0,985 η2 < 0,001 f < 0,001
Symptom-Related Outcomes									
Dyspnea (modified Borg scale)	2,00 ± 2,44	1,13 ± 2,04	*p* = 0,**025**	2,53 ± 2,76	1,70 ± 2,03	*p* = 0,**031**	*p* = 0,**002** η2 = 0,149 f = 10,153	*p* = 0,315 η2 = 0,017 f = 1,025	*p* = 0,950 η2 < 0,001 f = 0,004
Fatigue (modified Borg scale)	3,40 ± 2,58	2,00 ± 2,25	*p* = 0,**009**	3,33 ± 2,604	3,10 ± 2,18	*p* = 0,656	*p* = 0,**031** η2 = 0,078 f = 4,911	*p* = 0,309 η2 = 0,018 f = 1,055	*p* = 0,119 η2 = 0,041 f = 2,506
Incision pain (VAS)	4,50 ± 2,89	2,00 ± 1,98	*p* < 0,**001**	3,60 ± 2,29	2,53 ± 2,41	*p* = 0,082	*p* < 0,**001** η2 = 0,232 f = 17,529	*p* = 0,690 η2 = 0,003 f = 0,160	*p* = 0,098 η2 = 0,047 f = 2,831
Inflammatory Biomarkers									
CRP (mg/L)	141,14 ± 86,05	61,21 ± 59,51	*p* < 0,**001**	145,64 ± 75,05	83,84 ± 65,86	*p* < 0,**001**	*p* < 0,**001** η2 = 0,426 f = 42,985	*p* = 0,378 η2 = 0,013 f = 0,791	*p* = 0,405 η2 = 0,012 f = 0,703
NLR	10,20 ± 9,96	5,50 ± 3,33	*p* = 0,**006**	11,75 ± 9,28	5,48 ± 3,10	*p* < 0,**001**	*p* < 0,001 η2 = 0,276 f = 22,089	*p* = 0,598 η2 = 0,005 f = 0,281	*p* = 0,505 η2 = 0,008 f = 0,450
Albumin (g/dL)	6,17 ± 8,43	13,64 ± 33,27	*p* = 0,220	7,86 ± 10,75	13,93 ± 33,29	*p* = 0,318	*p* = 0,117 η2 = 0,042 f = 2,528	*p* = 0,833 η2 = 0,001 f = 0,045	*p* = 0,870 η2 < 0,001 f = 0,027

Data are presented as mean± standard deviation

*SBP* Systolic blood pressure, *DBP* Diastolic blood pressure, *HR*_*res*t_ Resting heart rate, *SpO2* Oxygen saturation, *RR* Respiratory rate, *VAS* Visual Analog Scale, *CRP* C-Reactive Protein; NLR, Neutrophil-to-lymphocyte ratio

significance at *p *< 0,05

*Repeated-measures ANOVA

**Mixed ANOVA

In intragroup comparisons, among the clinical parameters, significant changes were observed in heart rate and oxygen saturation in the older group. Heart rate decreased significantly from 91.53 ± 16.77 to 84.93 ± 15.24 beats/min (*p* = 0.023), and oxygen saturation increased from 94.30 ± 2.84% to 95.37 ± 2.39% (*p* = 0.015). No significant changes were observed in systolic blood pressure, diastolic blood pressure, or respiratory rate in either group.

Regarding symptom-related outcomes, dyspnea improved significantly in both groups, decreasing from 2.53 ± 2.76 to 1.70 ± 2.03 in the older group (*p* = 0.031) and from 2.00 ± 2.44 to 1.13 ± 2.04 in the middle-aged group (*p* = 0.025).

Fatigue scores improved significantly only in the middle-aged group, decreasing from 3.40 ± 2.58 to 2.00 ± 2.25 (*p* = 0.009), while no significant change was observed in the older group (*p* = 0.656). Similarly, incisional pain assessed using VAS decreased significantly only in the middle-aged group, from 4.50 ± 2.89 to 2.00 ± 1.98 (*p* < 0.001).

For inflammatory biomarkers, CRP levels decreased significantly in both groups, from 145.64 ± 75.05 to 83.84 ± 65.86 mg/L in the older group and from 141.14 ± 86.05 to 61.21 ± 59.51 mg/L in the middle-aged group (both *p* < 0.001). Likewise, NLR values decreased significantly from 11.75 ± 9.28 to 5.48 ± 3.10 in the older group (*p* < 0.001) and from 10.20 ± 9.96 to 5.50 ± 3.33 in the middle-aged group (*p* = 0.006). No significant changes were observed in albumin levels in either group.

## Discussion

In this ambispective, single-arm observational cohort study examining the age-related effects of the CIPT program on length of stay, clinical parameters, and inflammatory biomarkers in patients undergoing lung cancer surgery, no significant differences were found between middle-aged and older patients in the evaluated outcomes. However, statistically significant changes over time were observed in HR_rest_, oxygen saturation, and dyspnea in the older group, while significant improvements in dyspnea, fatigue, and incisional pain were observed in the middle-aged group compared with baseline. In addition, CRP and NLR levels decreased significantly in both groups compared with baseline values.

Hospital length of stay is a commonly used indicator for assessing recovery after lung cancer surgery. Perioperative interventions should be structured to minimize the impact of surgery and facilitate the patient’s return to daily life as early as possible [19, 20]. Prolonged hospitalization not only increases medical costs but also elevates the risk of infections, potentially leading to a decline in patients’ quality of life [21].

LOS is influenced by multiple factors. Advanced age and a higher number of comorbidities have been associated with prolonged hospital stays, likely due to slower postoperative recovery [22, 23]. In contrast, early mobilization has been reported to be protective against prolonged LoS by preventing complications such as venous thromboembolism, pneumonia, and muscle atrophy [24]. Additionally, surgical approach, duration of intensive care unit stay, postoperative physiotherapy-based interventions, early extubation, body mass index, male sex, and forced expiratory volume in 1 s values are among the other parameters that may influence LoS [25, 26].

A review of the literature reveals considerable variability in LoS following lung cancer surgery. In the United States, the average LoS after lung cancer surgery is reported to be approximately 4–5 days, and recent data suggest a gradual decline in LoS over the last decade [21, 27]. In contrast, another study reported a mean total LoS of 11.86 days [28]. Similarly, large database analyses have demonstrated substantial variability in LoS even among patients with uncomplicated postoperative courses. Giambrone et al. reported substantial variability in LoS after pulmonary lobectomy despite the absence of complications, reporting a median LoS of 4 days, and highlighting that LoS is influenced not only by surgical outcomes but also by patient-related and system-related factors such as age, sex, comorbidity burden, payer status, surgical approach, and hospital volume [27]. In a retrospective single-center study evaluating postoperative LoS after lobectomy for non-small cell lung cancer, Tat Bang et al. reported a median LoS of 5.2 days and demonstrated that perioperative factors such as early extubation, multimodal analgesia, and both preoperative and early postoperative physical therapy were associated with shorter hospitalization [25].

In addition to surgical and patient-related factors, perioperative rehabilitation strategies have also been investigated for their potential to enhance recovery after lung cancer surgery. Çınar et al. reported that a postoperative respiratory physiotherapy program significantly shortened hospital stays in patients undergoing lobectomy via thoracotomy [29]. In another randomized controlled trial, Jonsson et al. demonstrated that in-hospital physiotherapy including mobilization and breathing exercises increased early postoperative physical activity after lung cancer surgery; however, the intervention did not lead to a reduction in hospital length of stay [21]. Arbane et al. investigated the effect of an early postoperative strength and mobility training program following thoracotomy and found no significant difference in hospital length of stay between groups; mean LoS was 8.9 days in the intervention group and 11.0 days in the control group [30]. Nevertheless, given the multifactorial nature of hospital length of stay, the effects of rehabilitation-based interventions on this outcome may not always be fully reflected in LoS.

In our study, following the implementation of the CIPT program, the median LoS was 7 [5–10] days in the middle-aged group and 7 [6–10] days in the older group. Considering the considerable variability in reported hospital LoS after lung cancer surgery, the LoS observed in our cohort remained within a moderate range. In our sample, surgical procedures included both open thoracotomy and VATS, with approximately half of the patients undergoing each approach. Given that minimally invasive techniques such as VATS are generally associated with reduced surgical trauma and faster recovery compared with open thoracotomy, the observed LoS values may reflect the mixed surgical approach used in our cohort [11, 31].

In our study, comparison of LoS between the middle-aged and older groups revealed no statistically significant difference. Although advanced age and a higher burden of comorbidities are generally associated with prolonged hospitalization, our findings suggest that age alone may not be a major determining factor for postoperative LoS among patients receiving a structured CIPT program.

From an economic perspective, prolonged hospitalization may substantially increase the financial burden of lung cancer surgery, as the average cost of a single inpatient day has been estimated at approximately $2,200 [32]. In our cohort, despite the higher comorbidity burden in the older group, LoS did not differ between age groups, and the median discharge at 7 days suggests that the CIPT pathway may help maintain comparable LoS across age groups despite differences in comorbidity burden. Although a formal cost-effectiveness analysis was not performed, maintaining comparable LoS in a more comorbid older population may have favorable implications for healthcare resource utilization and hospital costs.

In addition to LoS, clinical parameters were evaluated to examine postoperative physiological changes observed during the CIPT program. None of the clinical parameters differed significantly between the middle-aged and older groups. Among the measured variables, SBP, DBP, HR_rest_, oxygen saturation, and RR, significant improvements in HR_rest_ and oxygen saturation were observed in the older group following the CIPT program. Although these changes were not statistically significant in the middle-aged group, a generally favorable trend was observed in both groups during the postoperative rehabilitation period. These findings suggest that the CIPT protocol can be safely implemented with respect to hemodynamic responses regardless of patient age. Moreover, despite a significantly higher number of comorbidities in the older group, no adverse events or clinical instability were observed during the program. These findings further support the safety and feasibility of early rehabilitation programs in the older patient population.

Another set of clinical parameters evaluated in our study included symptom-related outcomes such as dyspnea, fatigue, and incisional pain. Symptom burden has been shown to have a significant impact on patients undergoing lung cancer surgery and may adversely affect quality of life in this population [33]. In particular, symptoms such as dyspnea, fatigue, and incisional pain are known to negatively influence patients’ functional status [34]. Acute postoperative pain is especially common and typically increases in the early postoperative period compared with preoperative levels [35]. Pain is also a notable parameter due to its association with prolonged hospital stay and increased healthcare costs [36]. Previous studies have reported positive effects of different outpatient exercise-based rehabilitation programs on dyspnea, fatigue, and pain in patients undergoing lung cancer surgery [37, 38]. However, evidence regarding the impact of inpatient exercise programs during the early postoperative period and their influence on outcomes remains limited [21]. Nevertheless, Quist et al. reported that even rehabilitation initiated as early as 14 days after surgery was associated with a more favorable fatigue trajectory compared with later initiation, supporting the potential value of early postoperative rehabilitation in managing symptom burden. Taken together, these findings highlight the potential importance of initiating rehabilitation early in the postoperative period to better manage symptom burden after lung cancer surgery.

In our study, significant improvements in dyspnea, fatigue, and incisional pain scores were observed in the middle-aged group following the CIPT program compared with baseline. In the older group, however, a significant improvement was observed only in dyspnea, whereas fatigue and incisional pain did not change significantly. The absence of between-group differences may suggest that the CIPT program may contribute to maintaining symptom stability during the natural postoperative recovery period regardless of patient age. To our knowledge, few studies have specifically examined age-related differences in symptom response to early inpatient physiotherapy following lung cancer surgery, making direct comparison with previous literature difficult. From a clinical perspective, these findings may indicate that structured early rehabilitation programs such as CIPT can be safely implemented across different age groups without worsening postoperative symptom burden, even in older patients with a higher comorbidity load.

In addition to the clinical and symptom-related outcomes, inflammatory biomarkers were examined to better understand the postoperative inflammatory response. Following lung cancer surgery, an acute systemic inflammatory response is expected, typically characterized by elevated inflammatory markers such as CRP and NLR in the early postoperative period followed by a gradual decline as recovery progresses [39, 40], while albumin levels tend to increase as the inflammatory state resolves [41]. Studies evaluating the effects of postoperative comprehensive rehabilitation programs on inflammatory biomarkers following lung cancer surgery remain limited [42]. To our knowledge, no studies have specifically investigated age-related differences in these biomarker trajectories in this context. In the present study, the trajectories of these biomarkers generally followed the expected postoperative pattern in both age groups. Significant reductions in CRP and NLR levels were observed in both the middle-aged and older groups following the intervention. Although albumin levels did not show a statistically significant increase, a gradual upward trend was observed in both groups over time, consistent with the expected recovery process after lung cancer surgery. Importantly, no significant differences were found between the age groups. These findings suggest that the inflammatory recovery process progressed in a comparable manner in both groups regardless of age. Rather than indicating a direct causal effect of the exercise intervention on inflammatory biomarkers, our results may indicate that the CIPT program supported postoperative recovery without adversely affecting the expected inflammatory trajectory following lung cancer surgery.

### Limitations

This study has several limitations that should be considered when interpreting the findings. First, the absence of follow-up beyond the inpatient rehabilitation period limits the ability to determine whether the observed age-related patterns persist after hospital discharge. Second, although hospital length of stay has important economic implications, a formal cost-effectiveness analysis was not performed; therefore, the economic impact of the CIPT program could not be objectively evaluated. Third, oncological treatment-related variables such as chemotherapy regimens, immunotherapy, and other medication use were not analyzed in detail, although these factors may influence postoperative recovery, hospital length of stay, and clinical outcomes, particularly across different age groups.

Another limitation of this study is the absence of a non-intervention control group. Therefore, postoperative changes in clinical, symptom-related, and inflammatory parameters should be interpreted cautiously, as some of the observed changes may reflect the natural recovery process following lung cancer surgery. In addition, because all participants received the same structured postoperative physiotherapy pathway, the findings regarding length of stay should be interpreted in the context of age-group comparisons among patients receiving similar postoperative care.

Furthermore, patients requiring secondary ICU admission due to postoperative complications were excluded, as such events may substantially influence clinical, inflammatory, and hospitalization outcomes. In addition, participation in the structured inpatient physiotherapy program required hemodynamic stability during the early postoperative period, which may limit the generalizability of the findings to patients with severe postoperative complications or clinical instability.

Finally, the CIPT program was not designed as a structured aerobic training protocol with predefined intensity targets. Exercise intensity was individualized according to patient tolerance and clinical response as part of routine postoperative care, rather than standardized objective measures such as heart rate reserve or session-level Borg ratings. Therefore, the influence of exercise intensity on symptom-related outcomes, particularly in older patients, could not be fully determined.

## Conclusion

In conclusion, the findings of this study suggest that the CIPT program can be safely and feasibly implemented in both middle-aged and older patients following lung cancer surgery. Despite a higher comorbidity burden in the older group, no significant differences were observed between age groups in hospital length of stay, clinical parameters, symptom-related outcomes, or inflammatory biomarkers. These findings suggest that age alone may not represent a barrier to participation in structured early inpatient rehabilitation programs. The comparable length of stay between groups may also have potential economic implications, as maintaining comparable hospitalization duration across age groups despite differences in comorbidity burden could contribute to more efficient healthcare resource utilization. Nevertheless, given the absence of a non-intervention control group, the observed findings should be interpreted within the context of age-group comparisons among patients receiving similar postoperative rehabilitation.

## Data Availability

The datasets used and/or analysed during the current study are available from the corresponding author on reasonable request.

## Abbreviations

CIPT: Comprehensive in-patient physiotherapy
CRP: C-reactive protein
DBP: Diastolic blood pressure
HR_rest_: Resting heart rate
ICU: Intensive care unit
LoS: Length of stay
NLR: Neutrophil-to-lymphocyte ratio
PPP: Postoperative physiotherapy programs
ROM: Range-of-motion
RR: Respiratory rate
SBP: Systolic blood pressure
VAS: Visual analog scale
VATS: Video-assisted thoracoscopic surgery
